# Poly-ADP ribose polymerase (PARP) inhibitor regimens for platinum-sensitive ovarian cancer in randomized, double-blind, phase III controlled trials: protocol for a systematic review and network meta-analysis

**DOI:** 10.3389/fmed.2025.1539880

**Published:** 2025-01-23

**Authors:** Xiaolian Peng, Jie Liu

**Affiliations:** ^1^Department of Obstetrics and Gynecology, Xiegang Branch, Dongguan Municipal People’s Hospital, Guang Dong Province, China; ^2^Department of Vascular and Endovascular Surgery, Chinese PLA General Hospital, Beijing, China

**Keywords:** PARP inhibitor, ovarian cancer, Randomized Controlled Trial, phase III, protocol, network meta-analysis

## Abstract

**Introduction:**

Clinical trials have shown that PARP inhibitors are effective in treating patients with platinum-sensitive ovarian cancer. They have been indicated to improve progression-free survival or overall survival in patients with patients with platinum-sensitive ovarian cancer. However, there is insufficient comprehensive evidence regarding the comparison of different agents. To evaluate and compare the efficacy and side effects of various PARP inhibitors.

**Methods:**

We plan to conduct a network meta-analysis that includes randomized, double-blind, controlled phase III trials of Niraparib, Rucaparib, Olaparib, or Veliparib in patients with Platinum-sensitive ovarian cancer. The primary outcomes will be progression-free survival or overall survival. The secondary outcome will be grade ≥ 3 of treatment-emergent adverse events. Published and unpublished studies will be retrieved through PubMed, Embase, the Cochrane Library, ClinicalTrials.gov, and the World Health Organization (WHO) International Clinical Trials Registry Platform from 1990 to 2023. We will use STATA V.14.0 to perform all analyses, and the RevMan software to report the risk of bias in the included studies. We will determine the quality of evidence using the GRADEpro GDT software online version. This is a protocol description only. Results and conclusions are subject to completion. This study will be based on published studies, since no primary data collection will be carried out, no formal ethical assessment is required. The network graph and meta-analysis will be used to compare all PARP inhibitors. Their ranking will employ a rankogram, surface under the cumulative ranking curves, and mean ranks.

**Discussion:**

Our study will answer the most important question in platinum-sensitive ovarian cancer: which PARPi should be preferred regarding efficacy and side effects? Trials of platinum-resistant or refractory ovarian cancer will be excluded. The limitation is that the results of network meta-analyses do not yet have the same level of evidence as direct head-to-head trials. However, it is a useful complementary method when direct comparative studies cannot be performed. We plan to publish the results of this systematic review and network meta-analysis in peer-reviewed scientific journals, conferences, and the mass media.

**Systematic review registration:**

PROSPERO, CRD42024511248, available from: https://www.crd.york.ac.uk/prospero/display_record.php?ID=CRD42024511248.

## Introduction

### Rationale

Ovarian cancer is still one of the most difficult malignancies to treat, with 313,959 new cases and 207,252 cancer-related deaths each year worldwide (1). Epithelial ovarian cancer is the second most common reason for death among women with gynecological cancers, approximately 82% of patients with more advanced ovarian cancer will experience a relapse, and 60% of these will be platinum-sensitive (2). Although new anti-angiogenic therapies and poly (ADP-ribose) polymerase (PARP) inhibitor (PARPi) have dramatically improved outcomes for patients with ovarian cancer, there is still a need to understand better how to administer these regimens most effectively. This is particularly true in the absence of BRCA mutations and homologous recombination deficiency (HRD). About 20% of high-grade serous ovarian cancer (HGSOC) have mutations in the BRCA1/2 genes, this is linked to the high-fidelity HR DNA repair pathway (3). An increase in mutational load in tumor cells, which correlates with the anti-tumor immune response, has been reported with Olaparib. Anti-VEGF treatment may normalize the intratumoral vascular structure associated with the pathological response by reprogramming the immune microenvironment (4). 2005–2006, PARPi was first identified as highly efficacious against HR-deficient cancers (5). In 2009, a first-in-man clinical trial with Olaparib validated the synthetic lethal interaction between PARPi and BRCA1/BRCA2 deficiency (6).

The ovarian cancer treatment landscape changed in 2014 with the first approval of PARPi. These agents exploit BRCA mutations and DNA damage response (DDR) deficiencies. PARPi leads to the proliferation of single-stranded DNA breaks and the accumulation of double-stranded breaks. These breaks must be repaired by homologous recombination (HR) repair mechanisms (7). In the platinum-sensitive relapsed setting initial approvals for PARPi maintenance were limited to Olaparib for use in ovarian cancers with BRCA mutations. Subsequent data identified benefits in all subgroups and supported an extended scope for PARPi use (8). Between December 2014 and July 2017, Olaparib, Rucaparib, and Niraparib were approved for the treatment of recurrent ovarian cancer (5). On 19 December 2018, the Food and Drug Administration (FDA) approved Olaparib monotherapy for the first-line maintenance treatment of BRCA-mutated advanced ovarian cancer based on the results of the SOLO-1 trial. In addition, on 8 May 2020, based on the results of PAOLA-1, Olaparib was approved in combination with Bevacizumab for the first-line maintenance treatment of HRD-positive advanced ovarian cancer (9–11). Now many phase III randomized clinical studies of Niraparib (12), Rucaparib (13) Olaparib, and Veliparib (14) used for platinum-sensitive ovarian cancer have been published. Network meta-analysis has been used to extend conventional meta-analyses of multiple treatments for a given condition. Ranking of interventions using rank probabilities and rankograms is an attractive feature of network meta-analysis (15). However, comprehensive evidence comparing different PARPis based on randomized, double-blind, phase III controlled trials is lacking.

### Objectives

To evaluate and compare the efficacy and safety of four PARPis using network meta-analysis in patients with platinum-sensitive ovarian cancer.

## Methods

### Design and registration

We will conduct a network meta-analysis of randomized, double-blind, controlled phase III trials. This study’s protocol was registered with PROSPERO, registration number CRD42024511248. We will report our protocol according to the PRISMA-P 2015 checklist and the PRISMA Extension Statement for Reporting of Systematic Reviews (15).

### Eligibility criteria

The inclusion criteria of this network meta-analysis will be organized according to the acronym PICOS (16).

(P) Types of participants: all patients undergoing PARPi treatment in platinum-sensitive ovarian cancer.
(I) Intervention types: Four PARPis (Niraparib, Rucaparib, Olaparib (17), or Veliparib) at any dose and for any duration after primary maintenance or relapse.
(C) Comparison between interventions: all possible comparisons between the included PARPi, placebo, and Bevacizumab.
(O) Type of outcome measure: progression-free survival (PFS), overall survival (OS), or treatment-emergent adverse events (TEAEs) grade ≥ 3.
(S) Study type: only randomized, double-blind, controlled phase III trials will be included. No studies will be excluded based on language, publication date, or publication status.

### Information sources

We will search the following electronic databases: Cochrane Library (CENTRAL), MEDLINE via PubMed, Embase, ClinicalTrials.gov, and the World Health Organization’s (WHO) International Clinical Trials Registry Platform (ICTRP) from January 1, 1990, to December 16, 2023.

### Search strategy

We will identify all published, unpublished, and ongoing RCTs of different PARPi treatments in epithelial ovarian cancer. We will use the following search terms: Niraparib [mh] * OR Olaparib [mh] * OR Rucaparib [mh] * OR Veliparib [mh] * OR PARP inhibitors [mh] * ovarian cancer [mh] * Randomized Controlled Trial [pt] * OR Drug Therapies * (Table 1 showed a partial search strategy).

**TABLE 1:** Search strategy (from 1990/01/01 to 2023/12/16).

Database	Step	Search algorithm	Items found
PubMed	#1	(Niraparib [mh]) OR (2-(4-(piperidine-3-yl)phenyl)-2H-indazole-7-carboxamide) OR (niraparib hydrochloride) OR Zejula OR MK 4827)OR MK4827	603
	#2	(Olaparib [mh]) OR (AZD 2281) OR AZD2281 OR AZD-2281 OR AZD221 OR Lynparza OR AZD7648 OR AZD-7648 OR (7-methyl-2-((7- methyl-(1,2,4)triazole(1,5-a)pyridine-6-yl)amino)-9-(oxen-4-yl)purine-8-one) OR (7-methyl-2-((7-methyl(1,2,4) triazole(1,5-a)pyridine-6-yl)amino) 9-(tetrahydro-2 H-pyran-4-yl)-7,9-dihydro-8 H-purine-8-one)	2978
	#3	(Rucaparib [mh]) OR (PF-01367338) OR Rubraca OR (AG 014699) OR AG014699 OR AG-014699	616
	#4	(Veliparib [mh]) OR ((R)-2-methyl pyrrolidine-2-yl)-1H-benzimidazole-4-carboxamide) OR (2-(2-methyl pyrrolidine-2-yl)-1H-benzimidazole-4-carboxamide) OR (ABT 888) OR ABT888 OR ABT-888	597
	#5	(PARP inhibitors [mh])OR Inhibitors of Poly(ADP-ribose) Polymerase OR (PARP Inhibitor) OR (Inhibitor, PARP) OR (Poly(ADP-ribosylation) Inhibitors) PARP Inhibitors) OR (Inhibitors, PARP) OR (Inhibitors of Poly(ADP-ribose) Polymerases) OR (Poly(ADP-Ribose) Polymerase Inhibitor) OR (Poly(ADP-ribosylation) Inhibitor)	18,186
	#6	(ovarian cancer [mh]) OR (Neoplasm, Ovarian) OR (Ovarian Neoplasm) OR (Ovary Neoplasms) OR (Neoplasm, Ovary) OR (Neoplasms, Ovary) OR (Ovary Neoplasm) OR (Neoplasms, Ovarian) OR (Ovary Cancer) OR (Cancer, Ovary) OR (Cancers, Ovary) OR (Ovary Cancers) OR (Ovarian Cancer) OR (Cancer, Ovarian) OR (Cancers, Ovarian) OR (Ovarian Cancers) OR (Cancer of Ovary) OR (Cancer of the Ovary) OR (Carcinomas, Ovarian Epithelial) OR (Epithelial Carcinoma, Ovarian) OR (Epithelial Carcinomas, Ovarian) OR (Ovarian Epithelial Carcinomas) OR (Epithelial Ovarian Cancer) OR (Ovarian Epithelial Cancer) OR (Cancer, Ovarian Epithelial) OR (Cancers, Ovarian Epithelial) OR (Epithelial Cancer, Ovarian) OR (Epithelial Cancers, Ovarian) OR (Ovarian Epithelial Cancers) OR (Ovarian Cancer, Epithelial) OR (Cancer, Epithelial Ovarian) OR (Cancers, Epithelial Ovarian) OR (Epithelial Ovarian Cancers) OR (Ovarian Cancers, Epithelial) OR (Ovarian Epithelial Carcinoma) OR (Epithelial Ovarian Carcinoma) OR (Carcinoma, Epithelial Ovarian) OR (Carcinomas, Epithelial Ovarian) OR (Epithelial Ovarian Carcinomas) OR (Ovarian Carcinoma, Epithelial) OR (Ovarian Carcinomas, Epithelial) OR newly diagnosed Epithelial ovarian cancer OR Recurrent, platinum-sensitive Epithelial ovarian cancer OR Recurrent, platinum-resistant Epithelial ovarian cancer	127,326
	#7	#1 OR #2 OR #3 OR #4 OR #5 OR #6	127,326
	#8	Randomized Controlled Trial [pt] OR randomized controlled trial.mp	561,638
	#9	Controlled Clinical Trial[pt] OR Controlled Clinical trial.mp	622,505
	#10	Randomized[tiab] OR Randomized.mp	665,924
	#11	placebo[tiab]	226,969
	#12	drug therapy(sh) OR Therapy, Drug OR Drug Therapies OR Therapies, Drug OR Chemotherapy OR Chemotherapies OR Pharmacotherapy OR Pharmacotherapies	3,343,442
	#13	Randomly[tiab] OR Randomly.mp	405,385
	#14	Trial[tiab]	751,608
	#15	groups[tiab]	2,429,555
	#16	#8 OR #9 OR #10 OR #11 OR #12 OR #13 OR #14 OR #15	6,125,733
	#17	animals[mh] NOT humans[mh]	3,481,171
	#18	#16 NOT #17	5,333,234
	#19	#7 AND #18 (RCT)	1801

### Study records

#### Data management

Initial search records will be imported into ENDNOTE 20 literature management software.

#### Selection process

**FIGURE 1:**
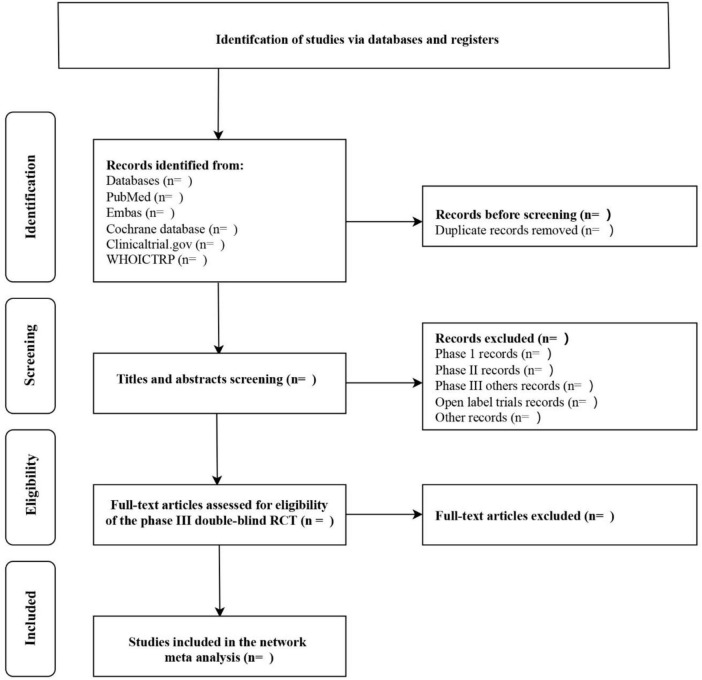
Flowchart of the literature selection process.

The titles and abstracts (if available) of all reports identified by the electronic searches were screened independently by two review authors (Xiaolian Peng and Jie Liu). Full texts were obtained for studies that appeared to meet inclusion criteria or for which title and abstract data were insufficient for clear adjudication. Full-text articles from all electronic sources and other search methods were independently assessed for inclusion criteria by two review authors (Xiaolian Peng and Jie Liu). Disagreement between the two review authors, if the problem cannot be solved properly, a third review author (Wentao Ni) will be consulted. Reasons for excluding studies after full-text searching will be recorded. All studies that meet the criteria will be included and analyzed effectively. For details, see Figure 1 Flowchart of the literature selection process.

#### Data collection process

To collect the data of interest, a standard data extraction form was created using Microsoft Excel 2016 (Microsoft Office Professional Enhanced Version 2016). Data from the included studies were extracted independently by the two authors (Xiaolian Peng and Liu Jie) using a predefined data extraction form. Dispute between the two review authors, if the problem cannot be solved appropriately, a third review author (Wentao Ni) will be referred to. If necessary, study authors were contacted for clarification or missing information. Acceptable selection and data extraction will be the responsibility of one reviewer (Xiaolian Peng) and will be subject to review by another reviewer (Jie Liu). Any conflicts will be resolved through discussion.

#### Data items

For each trial, the following data will be recorded: Methods: study name/design, title, journal, number of study centers and location, study setting, study duration (from the first enrollment of participants to the last follow-up), blinding, and sample sizes in intervention groups. Leading author, correspondence details, publication year, journal, duration. The number of treatment arms, the method of handling missing data, the randomization approaches, and the Jade score. Participant characteristics: the overall number, mean age, disease duration, comorbidities, the number in each arm, diagnostic method, type and the number of participants, cut-off level, risk factors of ovarian cancer, criteria for inclusion and exclusion, newly diagnosed, recurrent, complete response; partial response, BRCA mutation, HRD population. Interventions: type of PARPi, duration of treatment, frequency, dosage and main characteristics, follow-up, enrolled patients, previous lines of chemotherapy, previous bevacizumab use, the best response to the most recent platinum therapy, intention-to-treat (ITT), modified ITT (m-ITT), and Per-Protocol (PP) population. Outcomes: PFS, OS, TEAE. Results: the results for each outcome and study group, the number and percentages of outcome events, hazard ratios (HRs) with 95% CIs, *p*-values, and drop-outs. Adverse events: the number of patients who had adverse events, severity, and the number of patients who withdrew due to adverse events. Study funding: information about possible study funding. For details, see Table 2.

**TABLE 2:** Data items.

Data items	Specific content
Methods	Study name/design, title, number of study centers and location, study setting, study duration (from the first enrollment of participants to the last follow-up), blinding, and sample sizes in intervention groups. Leading author, correspondence details, publication year, journal, duration. The number of treatment arms, missing data methods, randomization approaches, and Jade score.
Participant characteristics	The overall number, mean age, disease duration, comorbidities, the number in each arm, diagnostic method, type and the number of participants, cut-off level, risk factors of ovarian cancer, criteria for inclusion and exclusion, newly diagnosed, recurrent, complete response; partial response, BRCA mutation, HRD population,
Interventions	type of PARPi, duration of treatment, frequency, dosage and main characteristics, follow-up, enrolled patients, previous lines of chemotherapy, previous bevacizumab use, the best response to the most recent platinum therapy, intention-to-treat (ITT), modified ITT (m-ITT), and Per-Protocol (PP) population,
Outcomes	progression-free survival (PFS), overall survival (OS), or treatment-emergent adverse event (TEAE)
Results	The results of each outcome and study group, the number and percentages of outcome events, hazard ratios (HRs) with 95% CIs, *p*-values, and drop-outs.
Adverse events	The number of patients who had adverse events, severity, or withdrew due to adverse events.
Study funding	Information about possible study funding.

#### Outcomes and prioritization

We define PFS as the time from the date of randomization to the first documented Response Evaluation Criteria in Solid Tumors (RECIST v1.1) progression or death from any cause, whichever occurs first. OS is the time from the date of randomization until death from any cause. All patients whose time of death was unknown at the time of analysis will be checked against the last date of record of survival. TEAEs are defined as all treatment-emergent adverse events (TEAEs) grade ≥ 3 with an initial date of on or after the date of the first dose of the study drug until the date of the last dose plus 28 days.

#### Risk of bias within individual studies

As part of the data extraction process, the risk of bias in the included studies was assessed by two review authors (Xiaolian Peng and Jie Liu) independently and in duplicate. Any conflict will be discussed between the same two review authors, if the problem cannot be satisfactorily resolved, a third review author (Wentao Ni) will be sought. Random sequence generation, allocation concealment, blinding of participants, blinding of outcome assessment, incomplete outcome data, selective reporting, and other biases will be assessed. The risk of bias for each trial will be independently assessed as low, unclear, or high, using the tool described in the Cochrane Collaboration Handbook as a reference (18).

We will contact the authors to obtain missing information if necessary. If none of the three areas is rated as high risk of bias and three or fewer areas are rated as unclear risk, the study is rated as low risk of bias overall. If one area is rated as a high risk of bias or none is rated as a high risk of bias but four or more are rated as unclear risk, the study is rated as moderate risk overall. All other studies are considered to be at high risk of bias overall (18). Each study’s overall risk of bias will be classified as above. We will report them using RevMan software (version 5.4.1, Copenhagen: The Nordic Cochrane Centre, The Cochrane Collaboration).

### Data synthesis

#### Description of the available evidence

We plan to conduct a network meta-analysis that includes randomized, double-blind, controlled phase III trials of Niraparib, Rucaparib, Olaparib, or Veliparib in adult patients with platinum-sensitive ovarian cancer. We first present the characteristics of the included studies. Then, we report on all proposed PARPi regimens and the results of each study.

#### Geometry of the network

Network plots will generated using STATA v.14.0 and R software v.4.2.1. Two-sided *p*-values are less than 0.05, which will be considered statistically significant. Network meta-analysis will likely include more studies and PARPis than traditional pairwise reviews (19, 20). We will use a network graph to summarize and compare the number of trials and patients of the different PARPis. The network graph includes nodes (points representing the competing PARPis) and edges (adjacent lines between nodes that indicate which PARPi was compared in the included trials). The amount of evidence for particular nodes and comparisons in the network graphs is represented by the size of the nodes, and the thickness of the edges. When comparing more than two PARPis, edges will sometimes be added to distinguish comparisons that may be part of multi-group trials. When 3 or more PARPis are connected through a polygon, a closed loop will be presented in the network (15).

### Network meta-analysis

#### Assessment of heterogeneity, transitivity, and inconsistency

##### Assessment of heterogeneity (pairwise meta-analysis)

We will perform pairwise meta-analyses for the pooled hazard ratio (95% CI), and heterogeneity will be assessed using the Cochran Q test, inconsistency index (I^2^ test), and meta-regression (18–20). I^2^ values of 25%, 50%, and 75% indicate low, moderate, and high levels of inconsistency, respectively (21). If heterogeneity is low, we will choose the model with fixed effects; if it is not, we will choose the following methods to deal with it: (1) Verification of the original data and the accuracy of the data extractive method. (2) Performing heterogeneity analysis through subgroup analyses and meta-regression. (3) Conduct sensitivity analysis to determine which studies caused the heterogeneity (22).

##### Assessment of transitivity

The transitivity assumption, also called similarity’, implies that studies comparing different interventions are sufficiently similar to make possible indirect comparisons (i.e., comparing two interventions via a third). We will assess the distribution of possible effect modifiers across all direct comparisons before conducting NMA to detect potential intransitivity (23–25). We will only include trials in patients with platinum-sensitive ovarian cancer; trials in patients with platinum-resistant or platinum-refractory ovarian cancer will be excluded. For this reason, it is assumed that patients in eligible trials have the same chance of being randomly assigned to each treatment (i.e., the transitivity assumption). The transitivity assumption is essential for valid indirect comparisons and will be further explored by looking at the distribution of potential effect modifiers across the different treatment comparisons (26, 27). For example, the platinum sensitivity definition, histological type included only high-grade serous ovarian cancer (including primary peritoneal or fallopian tube cancer) or high-grade endometrial cancer, age ≥18 years; randomized, double-blind, controlled phase III trials were included.

##### Assessment of inconsistency

The inconsistency assumption, namely the degree of disagreement between direct and indirect estimates, will be assessed using global and local methods. In addition, to test for design inconsistency across the network, we will consider a design-by-treatment interaction model (28–31). The local inconsistency will be evaluated using the node-splitting method (32–34). If there are discrepancies between direct and indirect results, subgroup analyses, sensitivity analyses, or meta-regression will be used to find the source of the discrepancy (35). We will not report the results of an NMA if there are significant unexplained inconsistencies (23). A consistency mode will be performed when the network meta-analysis contains closed loops. If the consistency test is passed, it suggests that the treatment effect from the direct evidence is consistent with the indirect evidence (36).

##### Ranking of competing PFS, OS, or TEAE

We will use a network meta-analysis to compare PFS, OS, or TEAE for all PARPis. The rankogram, surface under the cumulative ranking curves (SUCRA), and mean ranks will be used to estimate the ranking of different PARPis (37). A superiority index will be used to rank the cluster rank plot of risk estimates for PFS, or TEAE.

### Subgroup and sensitivity analyses

In the subgroup and sensitivity analyses, we examine the effect of study-level characteristics to investigate heterogeneity. Subgroup analyses will be performed as follows: (1) best response to the most recent platinum therapy, (2) previous use of bevacizumab, (3) previous lines of chemotherapy, (4) newly diagnosed or recurrent populations, (5) BRCA mutation status. (6) HRD status. Sensitivity analyses will include excluding small studies and bevacizumab use.

### Meta-bias (es)

#### Assessment of publication bias and small study effect

Comparison-adjusted funnel plots will be used to analyze publication bias. To assess whether small studies report higher effect estimates than larger studies in the pairwise meta-analysis (due to publication bias/small study effect), we will evaluate small study effects using funnel plots and Egger’s test for each outcome. Two independent reviewers (Xiaolian Peng and Jie Liu) will analyze and screen the risk of bias, imprecision, inconsistency, indirectness, publication bias, and large effect size.

### Confidence in cumulative evidence

#### GRADE quality assessment

The GRAD Epro GDT software online version will be used to assess the quality of evidence from direct, indirect, and network meta-analyses (37, 38). It includes assessing publication bias between studies, selective reporting within studies, and the strength of the body of evidence. The full text will be reported according to the PRISMA extension statement (15).

## Discussion

This NMA will evaluate the efficacy and safety of PARPis in platinum-sensitive ovarian cancer. In addition, this study will provide further stratified information on PFS, OS, or TEAE. This clinically relevant information may facilitate understanding the benefit/risk profile of PARPi. Our study will answer the most important question in platinum-sensitive ovarian cancer: which PARPi should be preferred regarding efficacy and side effects? We will use a novel approach to combine the results of trials in newly diagnosed and recurrent patients. This will allow NMA to include all related studies. Trials of platinum-resistant or refractory ovarian cancer will be excluded. The limitation is that the results of network meta-analyses do not yet have the same level of evidence as direct head-to-head trials. However, it is a useful complementary method when direct comparative studies cannot be carried out. Hopefully, our results will help clinicians make decisions on evidence-based treatment. They can also help update guidelines and design future randomized trial protocols.
